# Dysphagia Phenotypes in Spinal Muscular Atrophy: The Past, Present, and Promise for the Future

**DOI:** 10.1044/2021_AJSLP-20-00217

**Published:** 2021-04-06

**Authors:** Katlyn Elizabeth McGrattan, Robert J. Graham, Christine J. DiDonato, Basil T. Darras

**Affiliations:** a Department of Speech-Language-Hearing Science, University of Minnesota, Minneapolis; b Department of Rehabilitation, Masonic Children's Hospital, Minneapolis, MN; c Department of Anesthesiology, Critical Care, and Pain Medicine, Boston Children's Hospital, MA; d Department of Pediatrics, Northwestern University, Chicago, IL; e Human Molecular Genetics and Physiology Program, Anne & Robert H. Lurie Children's Hospital, Chicago, IL; f Department of Neurology, Boston Children's Hospital, MA

## Abstract

**Purpose:**

The aim of this study was to provide clinicians with an overview of literature relating to dysphagia in spinal muscular atrophy (SMA) to guide assessment and treatment.

**Method:**

In this clinical focus article, we review literature published in Scopus and PubMed between 1990 and 2020 pertaining to dysphagia in SMA across the life span. Original research articles that were published in English were included. Searches were conducted within four themes of inquiry: (a) etiology and phenotypes, (b) respiratory systemic deficits and management, (c) characteristics of natural history dysphagia and its treatment, and (d) dysphagia outcomes with disease-modifying therapies. Articles for the first two themes were selected by content experts who identified the most salient articles that would provide clinicians foundational background knowledge about SMA. Articles for the third theme were identified using search terms, including *spinal muscular atrophy,* *swallow,* *dysphagia,* *bulbar, nutrition,* *g-tube,* *alternative nutrition,* *jaw,* *mouth,* *palate,* OR *mandible*. Search terms for the fourth theme included *spinal muscular atrophy* AND *nusinersen* OR *AVXS-101/onasemnogene abeparvovec-xioi*.

**Review of Pertinent Literature:**

Twenty-nine articles were identified. Findings across identified articles support the fact that patients with SMA who do not receive disease-modifying therapy exhibit clinically significant deficits in oropharyngeal swallow function. Few investigations provided systematic information regarding the underlying physiological deficits responsible for this loss in function, the timing of the degradation, or how disease-modifying therapies change these outcomes.

**Conclusion:**

Future research outlining the physiological and functional oropharyngeal swallowing deficits among patients with SMA who receive disease-modifying therapy is critical in developing standards of dysphagia care to guide clinicians.

Spinal muscular atrophy (SMA) is a progressive neuromuscular disorder that occurs in one out of every 11,000 live births (Sugarman et al., 2012). Clinical features of this condition span a wide severity continuum. In its most severe and prevalent form, infantile-onset Type I, previously healthy infants exhibit rapidly progressing weakness of the muscles that facilitate the basic life-sustaining functions of deglutition and respiration within the first 6 months of life. Deficits within these critical functions have historically been a leading source of infant morbidity and mortality: causing infants to aspirate secretions and nutrition into a pulmonary system incapable of aspirate expectoration (Birnkrant et al., 1998; Finkel, Weiner, et al., 2014; Grünebaum et al., 1981). Those with the mildest form, Type IV, may not experience symptoms until adulthood, at which time the resulting functional impairments are relatively mild and more focal to mouth opening (Russman, 2007; Wadman et al., 2014; Wang et al., 2007).

Until recently, the absence of treatments capable of halting this neuromuscular degeneration resulted in a single discipline dysphagia management approach in which the dysphagia expert's involvement, if any, was limited to a single palliative care visit for patients with SMA Type I. However, this landscape quickly changed starting in 2016 with the Food and Drug Administration (FDA) approval of the first disease-modifying therapy nusinersen enabling survival among the most fragile of the patients with SMA (Finkel et al., 2017). This changed the traditional palliative approach to dysphagia management to a rehabilitation approach due to the continued observation of swallowing deficits despite gross clinical improvements in other motor functions. Following this time, two more pharmaceuticals, such as the gene replacement therapy onasemnogene abeparvovec-xioi and the oral medication risdiplam, have been approved, and others are in testing (Mendell et al., 2017; Servais et al., 2020), with the current focus moving from survival to now developing regimens that will maximize swallowing and other quality of life outcomes. The shear complexity of these patients who are being treated with rapidly advancing therapeutic regimens, as well as the risks of being mismanaged, necessitates the rapid integration of dysphagia experts into the neuromuscular disorder team. The aim of this clinical focus article was to guide speech-language pathologists in their SMA dysphagia management by providing an overview of the supporting literature. This was accomplished by using a multidisciplinary clinical research team to review literature within four domains of inquiry: (a) etiology and phenotypes, (b) respiratory systemic deficits and management, (c) characteristics of natural history dysphagia and its treatment, and (d) dysphagia outcomes with disease-modifying therapies. Our clinical focus approach, which combines the most relevant SMA literature with the insight of multidisciplinary clinical experts, provides clinicians with the necessary time-sensitive guidance relating to the management of this underserved population while scientists work to accrue a greater breadth of evidence capable of enabling further clinical advances.

## Method

A multidisciplinary team of SMA clinical and scientific experts in deglutition (K. E. M.), neurology (B. T. D.), pulmonology (R. J. G.), and basic science-genetics (C. J. D.) was formulated to execute this clinical focus article. This multidisciplinary approach was taken due to the appreciation that adequate dysphagia management requires some high-level understanding of deficits and treatments within the interdependent body systems that influence swallowing abilities and outcomes. Four themes of inquiry were identified within this framework: (a) etiology and phenotypes, (b) respiratory systemic deficits and management, (c) characteristics of natural history dysphagia and its treatment, and (d) dysphagia outcomes with disease-modifying therapies. Articles within each theme of inquiry were identified through electronic searches of Scopus and PubMed databases. Inclusion criteria required an article to be original research or a case report that was published in English between 1990 and 2020. Secondary analyses of original research results were not included. Literature search and article selection for each theme of inquiry were completed by the associated content expert for that theme, with methodology further outlined below:

*Etiology and phenotypes* and *respiratory systemic deficits and management*: The aim of these themes of inquiry was to provide the reader with a high-level overview of the critical background information as a foundation to contextualize the dysphagia review results. In line with this goal, the content experts for these domains (B. T. D., R. J. G., C. J. D.) selected the most salient articles in these areas based on their expertise and used them to generate reviews of these areas.

*Characteristics of natural history dysphagia and its treatment*: Search terms for this field of inquiry included *spinal muscular atrophy* AND *swallow,* *dysphagia, bulbar, nutrition, g-tube, alternative nutrition, jaw, mouth, palate,* OR *mandible*. Results were reviewed by the deglutition expert (K. E. M.) for relevance of their content toward oropharyngeal swallowing anatomy, physiology, or function among patients with SMA who did not receive pharmaceutical disease-modifying therapy. Those articles whose relevance was in question based on title or abstract were reviewed in full. Articles relating to nutrition were only included if they discussed use of alternative nutrition. Reference lists of articles meeting the aforementioned criteria were reviewed to identify additional articles not identified in the initial search. Included articles were reviewed and categorized based on the type of swallowing outcomes they reported: alternative nutrition, patient-reported chewing/swallowing deficits, instrumental observations of oropharyngeal swallowing physiology, mandibular range of motion/strength, or craniofacial morphology/malocclusion.

*Dysphagia outcomes with disease-modifying therapies*: The search terms from the *characteristics of natural history dysphagia and its treatment* theme of inquiry did not identify all articles reporting results from the two FDA-approved disease-modifying therapy trials (nusinersen and AVXS-101). These articles were identified using the search terms *spinal muscular atrophy* AND *nusinersen* OR *AVXS-101/onasemnogene abeparvovec-xioi,* with inclusions of articles specific to original research clinical trials, with all secondary analyses of these trials excluded. Inclusion criteria and review processes from the *characteristics of natural history dysphagia and its treatment* were applied, with only those trials reporting on one of the dysphagia outcomes included in the review. Risdiplam, a brain-penetrant, orally bioavailable, small molecule that targets *SMN2* exon 7 splicing, recently received FDA approval. It is not included here, as original research clinical trial results have been orally presented, but not yet published.

## Review of Pertinent Literature

### Theme 1: Etiology and Phenotypic Clinical Presentation

**Figure 1.**
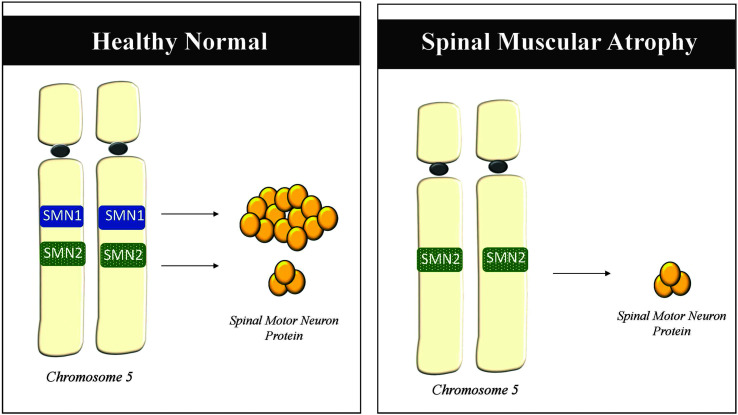
Healthy normal individuals have two copies of *SMN1* that produce the majority of functional spinal motor neuron protein necessary for motor neuron survival. Patients with SMA have mutations or deletions in both *SMN1* genes that inhibit sufficient production of functional spinal motor neuron protein.

SMA is an autosomal recessive neuromuscular disorder resulting from homozygous deletions and/or mutations of the *Survival Motor Neuron 1* (*SMN1*) gene on Chromosome 5 (Darras et al., 2017; Lefebvre et al., 1995; Markowitz et al., 2012; Yeo & Darras, 2020). As a result, both parents must have the *SMN1* deletion or mutation on at least one chromosome in their germ cells, and each pass along their missing or mutated *SMN1* gene for their child to develop SMA. The *SMN1* gene produces the SMN protein that plays a critical role in the function of the motor neurons in our brainstem and spinal cord to facilitate skeletal muscle movements necessary for functions such as respiration, ambulation, and deglutition. When SMN levels are low due to mutation or deletion of *SMN1,* this leads to motor neuron degeneration that ultimately results in weakness and progressive atrophy due to denervation, which is greatest in the proximal muscles, though sensation and cognition are spared. The severity of these symptoms is in part dependent on the amount of SMN protein produced by a nearly identical gene, *SMN2*. Although *SMN1* produces the majority of SMN protein, *SMN2* produces a small amount of functional protein that can sustain cellular function, at least partially, in patients with bi-allelic deletions or mutations in the *SMN1* gene (Lefebvre et al., 1995; Markowitz et al., 2012; Yeo & Darras, 2020). As a general result, individuals who have more *SMN2* copies frequently have a milder SMA phenotype and disease progression (see Figure 1; Calucho et al., 2018; Feldkotter et al., 2002). Table 1 provides a historic classification of SMA phenotypes (Munsat, 1991), which will also be discussed in more detail in the subsequent paragraph of the article.

**Table 1.** Historic classification of spinal muscular atrophy.

Type	Milestone	Age of onset	Life expectancy
0	Never sit unassisted	Prenatal	< 6 months
I	Never sit unassisted	0–6 months	< 2 years
II	Sit but cannot stand unassisted or walk	6–18 months	70% alive at 20 years
III	Sit, stand, and walk at some point	> 18 months	Normal
IV	Sit, stand, and walk	10–30 years	Normal

While the association between *SMN2* copy number and disease severity provides some predictive value into the anticipated severity of disease progression, it is far from a perfect predictive metric. Clinical SMA staging of disease severity has historically been based primarily on the maximum motor milestone the individual has achieved and secondarily on the age of symptom onset (see Table 1; Munsat, 1991). SMA Type 0, the most severe and rare form of SMA with fetal onset and diagnosis at birth, is typically associated with one copy of *SMN 2*. Clinical features at the time of birth include absent limb or face movement, including suckle response, areflexia, contractures, and need for mechanical ventilation (Finkel et al., 2015; Grotto et al., 2016; Matesanz et al., 2020). These deficits progress rapidly; with fatality frequently within weeks of birth. In contrast, SMA Type I is the most common form of SMA that accounts for more than 50% of SMA live births. This variant presents with one to three copies of *SMN2,* most frequently two copies (about 80%; Calucho et al., 2018). In this form, infants who previously were thought to be healthy by caregivers stop making gains on motor milestones and exhibit developmental regression within the first 6 months of life, which inhibits their ability to ever sit independently (Munsat, 1991). These infants, if left untreated, succumb to profound hypotonia and weakness due to motor neuron loss that impedes their ability to hold their head up or move their limbs. Some of the greatest motor neuron degeneration and loss is seen in those neurons that innervate the respiratory and swallowing musculature, which is so severely impacted that it has historically caused infant death by 2 years of age (Finkel, McDermott, et al., 2014). SMA Type II, a less severe form of SMA, does not present until 7–18 months of age, at which time the progression impedes the child's ability to walk independently, but these children can sit on their own. Though their life expectancies are reduced, the majority of patients will live into adulthood with much milder reductions in muscle weakness (Chung et al., 2004; Munsat, 1991). About 80% of patients with SMA Type II will have three copies of *SMN2* on genetic testing (Calucho et al., 2018; Feldkotter et al., 2002). SMA Type III has much higher variability in copy number, with the majority of patients having three or four copies of *SMN2* and tremendous variability in clinical presentation. Individuals with SMA Type III are diagnosed after 18 months of age and typically achieve all motor milestones, though it may require some ambulatory supports or lose ambulation as the disease progresses. In contrast, those with SMA Type IV are much more rare and do not manifest symptoms until adulthood (Darras et al., 2017; Munsat, 1991). Prior to 2016, there was no disease-modifying treatment for SMA. This resulted in a largely palliative therapeutic approach for patients with SMA Type I and, in less severe forms, the provision of supplemental respiratory and nutritional support to facilitate patient comfort and prolong quality of life throughout the inevitable neurodegeneration. Today, with three FDA-approved therapies that have clearly altered survival and disease trajectory (Finkel et al., 2017; Mendell et al., 2017; Servais et al., 2020) and with more in the pipeline, understanding respiratory deficits and dysphagia characteristics in the untreated state, as described below, creates a baseline to understand the underlying etiology of dysphagia and ways of mitigating risks in this new exciting treatment era.

### Theme 2: Respiratory System Deficits and Management

**Figure 2.**
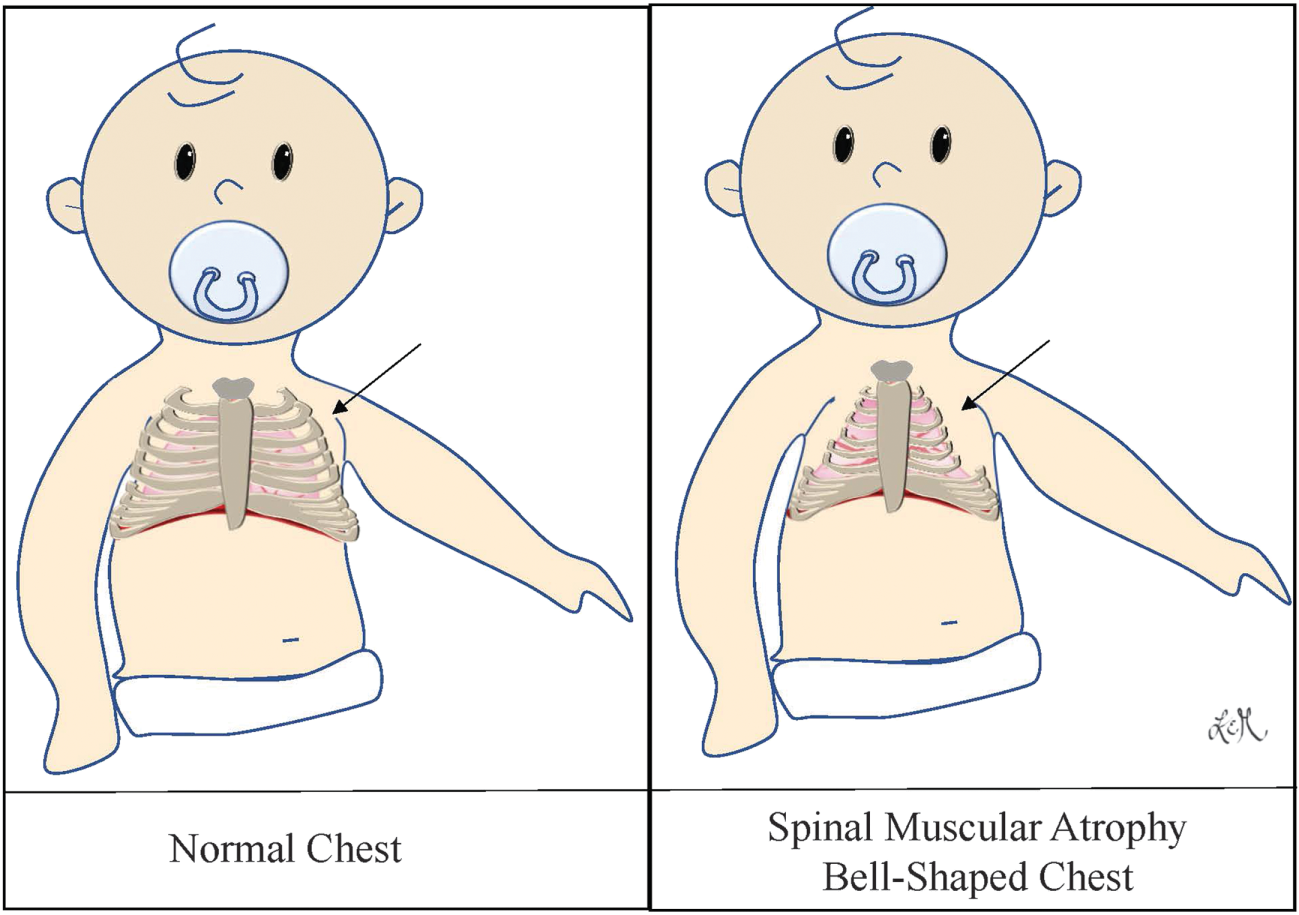
Bell-shaped chest resulting from insufficient intercostal muscle strength preventing against inward paradoxical rib cage prolapse during inspiration.

Chronic respiratory insufficiency, or respiratory failure, is a leading morbidity among children and adults with SMA. While neuromuscular insufficiency is the underlying etiology that belies all aspects of the respiratory complications, the primary drivers and manifestations for an individual patient at any given time are multifaceted, variable, and complicated (Bergofsky, 1979; Finkel, Weiner, et al., 2014). Individuals with SMA exhibit progressive weakness of their respiratory musculature, with the diaphragm generally last to be affected (Grychtol et al., 2018). Early in disease progression, weakness of the external intercostals inhibits full rib cage expansion and the generation of full tidal volumes during inspiration. These lung volumes are further reduced with disease progression by the inward paradoxical rib cage prolapse that occurs when the intercostals can no longer stabilize the rib cage against the force of the contracting diaphragm during inspiration (Grychtol et al., 2018). The immediate effect of this, in addition to a reduction in tidal volume, is the need to put forth greater effort to maintain cardiopulmonary stability (Grychtol et al., 2018). Long-term implications include chest wall deformities, such as the development of a “bell-shaped” chest that is wider at the bottom than at the top (see Figure 2). This, exacerbated frequently by severe scoliosis, causes further restrictions to chest wall expansion and optimization of natural chest recoil forces. Initial signs of respiratory insufficiency typically manifest as periods of hypoxia during sleep when respiratory musculature is lowest in tone (Bergofsky, 1979; Finkel, Weiner, et al., 2014; Grychtol et al., 2018). While not investigated to the authors' knowledge to date, these deficits have tremendous potential to further exacerbate physiological swallowing deficits that will be elucidated in subsequent sections by disrupting respiratory–swallow coordination.

Although the lung tissue itself is not directly affected by SMA, these deficits generally arise with disease progression as a result of the aforementioned impairments in respiratory mechanics coupled with micro- or macro-aspiration events. Weakness in the respiratory musculature inhibits cough strength and consequently clearance of airway pathogens such as airborne microorganisms and aspirated materials, including secretions, gastric contents, or ingested nutrition. This leads to micro-atelectasis, recurrent chest infections, and, over time, parenchymal lung disease, which inhibits gas exchange (Grychtol et al., 2018). Progression of these deficits commonly results in respiratory failure, which is more profound in infants with SMA Type I but is widely experienced by those classified with SMA Type II. It is important to consider that respiratory decompensation can be rapid, resulting from muscle weakness, or preceded by even the most minor respiratory infection. Older individuals with SMA Type III can also experience respiratory difficulties and aerodigestive compromise in the context of intercurrent illnesses, aspiration, surgeries, or pregnancy. As such, conservative approaches to dysphagia management have been critical in facilitating patient longevity, especially among the more severe forms of SMA, as there is tremendous risk that even the most trace aspiration event could lead to morbidity.

Prophylactic management of these respiratory deficits before symptom onset is now standard of care for individuals with SMA (Finkel et al., 2018). The most widely used prophylactic measures across all SMA severities are those that facilitate airway clearance. Manual chest physiotherapy combined with mechanical insufflation–exsufflation techniques (e.g., CoughAssist) are recommended immediately upon diagnosis among patients with SMA Types I–II and as needed with less severe forms (Finkel et al., 2018). Subsequent interventions to facilitate ventilation are typically withheld until weakness in respiratory musculature progresses and the first signs of respiratory insufficiency manifest. This includes the nocturnal use of noninvasive ventilation with bilevel positive airway pressure to promote lung inflation, prevent hypoxia, and prevent chest well malformations (Finkel et al., 2018; Grychtol et al., 2018). Continuous positive airway pressure is contraindicated in patients with SMA, as weak patients do not have the strength to exhale over the continuous pressure that persists throughout the expiratory phase of the respiratory cycle (Finkel et al., 2018; Grychtol et al., 2018). Due to the high risk for rapid respiratory decompensation, caregivers are often consulted to extend bilevel positive airway pressure use to daytime periods or increase inspiratory pressures in effort to prevent further decline, which will require intubation (Finkel et al., 2018; Grychtol et al., 2018). Despite the provision of noninvasive ventilation attempts, profound weakness in respiratory musculature, coupled with swallowing deficits that inhibit secretion management, can result in the need for tracheostomy placement to allow ventilation and pulmonary clearance (Grychtol et al., 2018). While all the aforementioned interventions are critical to patients with SMA's well-being, rigorous dysphagia assessment and treatment continue to be one of the most critical aspects of care for any affected individual. There are obvious implications for aspiration risk with subsequent reactive airway disease and pneumonias for those with more overt aerodigestive compromise. Yet, assessing and supporting swallowing capacity are essential to optimize growth and minimize calorie expenditure while maintaining hydration and nutrition.

### Theme 3: Characteristics of Natural History Dysphagia and Its Treatment

Results of the 23 included articles that were identified within this theme of inquiry are summarized in Table 2. Articles provided data pertaining to alternative nutrition needs and rates (seven articles), reports of functional deficits in chewing or swallowing (16 articles), measures of physiological swallowing deficits (eight articles), reductions in mandibular range of motion or strength (13 articles), and impairments in craniofacial structures or occlusion (six articles) across the SMA continuum.

**Table 2.** Details of investigations outlining dysphagia correlates in natural history of untreated patients with spinal muscular atrophy (SMA).

Authors	Year	SMA type	*N* ^a^	Alternative nutrition	Reported chewing/swallowing functional deficits^b^	Measured physiological swallowing deficits^c^	Mandibular range of motion/strength^d^	Craniofacial morphology/occlusion^d^
Banno et al.	2017	Not reported	111			X		
Birnkrant et al.	1998	I	4	X				
Cha et al.	2010	II	1		X	X	X	
Chen et al.	2012	II, III	108		X		X	
Choi et al.	2020	I	11	X	X	X	X	
Davis et al.	2014	I	44	X				
Durkin et al.	2008	I	12	X		X		
Granger et al.	1999	Not reported	16				X	
Grotto et al.	2016	0	16		X			X
Hashizume et al.	2017	Not reported	111			X		
Houston et al.	1994	Not reported	25					X
Kooi-van Es et al.	2020	I, II, III	13		X			
Messina et al.	2008	II	122	X	X		X	
Morris et al.	2020	II	1		X		X	
Suzukia et al.	2007	II	1		X	X	X	X
van Bruggen et al.	2011	II	12		X		X	X
van Bruggen et al.	2016	II, III	60		X		X	X
van den Engel-Hoek et al.	2008	II	1		X	X	X	X
van den Engel-Hoek et al.	2009	II	6		X	X	X	
van der Heul	2020	I	11	X	X			
Wadman et al.	2014	I, II, III, IV	145		X		X	
Willig et al.	1994	Not reported	38		X		X	
Yuan et al.	2017	I, II	7	X	X			

a Represents the number of natural history participants with SMA within a given investigation if other disease states or treatment arms were included.

b Included investigations reporting patient-reported outcomes formally acquired through patient questionnaires or clinical reports of problems without indication of instrumental assessment.

c Swallowing references instrumentally identified deficits in oral or pharyngeal sucking, chewing, or swallowing physiology.

d Included articles reporting explicit patient-reported outcomes of impairments in these areas or instrumental assessments.

In contrast to the large body of literature mapping the nature and progression of the various deficits in untreated SMA, much less is known regarding the correlates of dysphagia and its progression among historic patients with SMA. That which is known is primarily focused on the documentation of clinical symptoms and need for alternative nutrition. In addition to these reports, isolated investigations have revealed the underlying anatomic and physiological deficits responsible for these functional impairments. These include impairments in oropharyngeal swallowing, mandibular range of motion and strength, and craniofacial morphology. These deficits, like the aforementioned gross and fine motor deficits, span a wide spectrum of severity and prevalence correlated with SMA type and motor function (Chen et al., 2012), as described below.

*Oropharyngeal swallowing:* Deficits in swallowing have historically been the most profound and functionally debilitating impairment among patients with SMA. Though patients with SMA Type 0 are born with these profound sucking and swallowing deficits that warrant alternative nutrition, their need for mechanical ventilation at birth precludes their oropharyngeal physiology from being the primary source for their inability to eat (Grotto et al., 2016; Matesanz et al., 2020). Grotto et al. (2016) reported 100% patients with SMA Type 0 exhibited lingual fasciculations and impairments in suck/swallow reflexes at birth. Functional swallowing deficits are especially true for patients with SMA Type I. Deficits manifest as a rapid degradation in the infant's ability to safely and successfully orally feed and manage oral secretions as they progress throughout their first year of life. This results in a change from full oral intake without symptoms of impairment to complete dependence on alternative nutrition and hourly suctioning for secretion management over a period of 1.5–4 months from time of symptom onset (Birnkrant et al., 1998; Choi et al., 2020; Davis et al., 2014; Durkin et al., 2008; Finkel, McDermott, et al., 2014; Yuan et al., 2017). Choi et al. (2020) indicated swallowing typically began degradation around 6 months of age (range: 5–12), with Finkel, McDermott, et al. (2014) reporting 100% of infants with SMA Type I required feeding support by 12 months of age. What is most alarming about the underlying changes in physiology is that they often go unnoticed until the deficits are so severe they require infant hospitalization due to respiratory distress, pneumonia, poor weight gain, or dehydration (Birnkrant et al., 1998). These conditions pose disastrous implications for infants, as their already weak respiratory systems are frequently unable to recover from these disorders and are a frequent source for infant mortality. This inevitable swallowing degradation has led to the development of standard practice regimens among patients with SMA Type I, including the prophylactic placement of a gastrostomy tube and completion of Nissen fundoplication at the time of diagnosis (Durkin et al., 2008).

**Figure 3.**
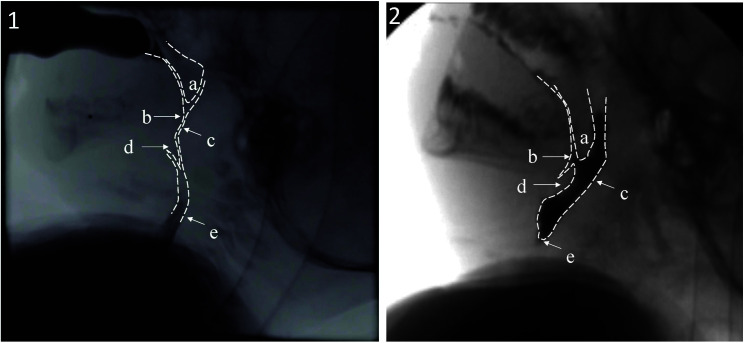
Fluoroscopic visualization of maximum pharyngeal contraction during swallowing in (1) a healthy normal infant and an (2) infant with spinal muscular atrophy 1. Infant with spinal muscular atrophy Type 1 has incomplete (a) soft palate elevation, (b) tongue base retraction, (c) pharyngeal stripping wave, (d) epiglottic inversion, and (e) upper esophageal segment opening resulting in no clearance of bolus from the pharynx (residue).

Despite the appreciated deficits in SMA Type I swallow function, there has been a paucity of research elucidating the underlying physiological correlates responsible for these changes. Available reports, isolated to case studies and small series reports using unvalidated fluoroscopic metrics, indicate generalized deficits in sucking and swallowing physiology, resulting in 100% of patients with SMA Type I aspirating on fluoroscopic evaluation (Durkin et al., 2008; Grünebaum et al., 1981). Consistent with reports of reduced lingual strength are findings of resting lingual fasciculations among 80%–100% of patients with SMA Type I (Choi et al., 2020; Wadman et al., 2014). Other reports have eluded to deficits in pharyngeal clearance resulting in pharyngeal residue (Choi et al., 2020). To fill this void and enable future investigations examining the effect of different SMA disease-modifying treatments, we developed an interdisciplinary team of clinicians and researchers from SMA centers worldwide to identify the physiological correlates for these functional changes. Preliminary findings indicate profound deficits in sucking efficiency, tongue base retraction, pharyngeal stripping wave, hyolaryngeal movement, and upper esophageal opening that contribute to inefficient bolus expression and oftentimes absent clearance of the bolus from the pharynx (K. McGrattan et al., 2019). These deficits led to aspiration of residue after the swallow in 92% of the sample (see Figure 3; K. McGrattan et al., 2019). Our findings, suggesting loss of cranial nerve function, are supported by postmortem studies that revealed pathological changes in cranial nerves among patients with SMA (Byers & Banker, 1961; Korinthenberg et al., 1997). Byers and Banker (1961) reported these changes were most severe in the motor nuclei of trigeminal, facial, and hypoglossal nerves, as well as nucleus ambiguous—some of the key oropharyngeal regulators.

Though not as severe or prevalent, swallowing impairments have also been reported in patients with less severe forms of SMA Types II–IV (Bartolome & Neumann, 1993; Kooi-van Es et al., 2020). As in other areas of motor function, the prevalence and severity of these deficits decreases with increasing SMA staging, though not *SMN2* copy number (Chen et al., 2012). Chen et al. (2012) found 62% of individuals with SMA Type II reported dysphagia, while only 23% of individuals with SMA Type III reported such impairments. Interestingly, investigators found the best predictor of dysphagia among these patients not to be SMA type, but the individual's current level of motor function as categorized as walker, sitter, or nonsitter status. Those with lower levels of function had a 7.6 greater odds of reporting dysphagia compared to those of higher status as demonstrated by reports of dysphagia among 11% of those patients who could currently stand compared to 54% of patients who could only sit (Chen et al., 2012).

In contrast to patients with SMA Type I who typically lose all oropharyngeal swallowing ability prior to initiating puree and solid intake, an equally high, if not greater, proportion of dysphagia symptoms among SMA Types II–IV relate to choking and chewing difficulties with solids (Chen et al., 2012; Messina et al., 2008). Among patients with SMA Types II and III, 17% self-report difficulty in swallowing liquids (Chen et al., 2012), while difficulty in chewing and swallowing solids, which often results in choking, was reported in 28% and 20% of patients, respectively (Messina et al., 2008). The slower disease progression among these less severe forms results in a much later manifestation of deficits, with fewer than 10% of patients demonstrating clinical impairments before 5 years of age, 23%–26% reporting these symptoms by 10 years of age, and nearly 50% by 20 years (Messina et al., 2008). These impairments not only pose functional barriers to eating but also appear to play a role in respiratory and nutritional deficits. These include aspiration pneumonia in 3%–9% of patients and poor weight gain in 33%–37% (Atsuta et al., 2006; Chen et al., 2012; Messina et al., 2008). Despite these high rates of poor nutrition, research suggests a smaller proportion of these patients will receive alternative nutrition (Messina et al., 2008).

It is important to note that, in contrast to patients with SMA Type I who often experience aspiration pneumonia–inducted morbidity after initial presentations of swallowing impairment, the rate of neuromuscular degradation among the less severe forms of SMA results in a much slower progression to severe dysphagia consequences. In an investigation of individuals with adult-onset SMA, Atsuta et al. (2006) reported that the average age of first aspiration pneumonia event among the 9% of individuals who experienced aspiration pneumonia was 62 years—8 years following the average age of first dysphagia symptoms. Likewise, at the time of study conclusion, incidence of aspiration-related death was 4% (Atsuta et al., 2006). While future investigations are necessary to clearly delineate these causal relationships and risk factors, these discrepancies in severity and timing of outcomes likely reflect differences in the amount and frequency of aspiration, the differences in patient mobility, and, of critical importance, the greater integrity of the respiratory system that enables the expectoration of aspirated material.

**Figure 4.**
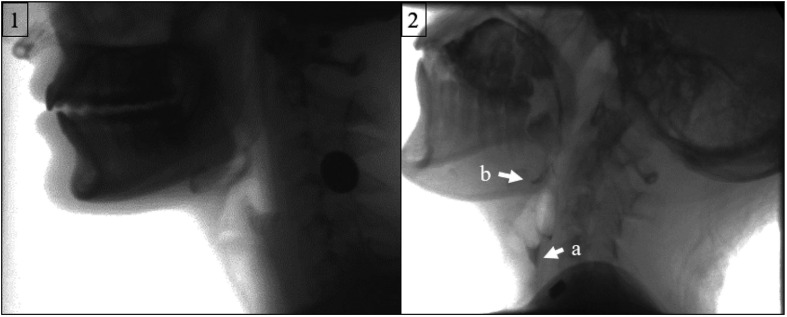
Fluoroscopic visualization of pharynx after the swallowing in (1) a healthy young adult and a (2) young adult with spinal muscular atrophy (SMA) Type III. Adult with SMA Type III has (a) pyriform and (b) vallecular residue, as indicated by arrows after the swallow, whereas the healthy normal adult has no segment opening resulting in no clearance of bolus from the pharynx (residue). Note the retracted neck posture in the patient with SMA.

Findings from fluoroscopic investigations examining the physiological correlates for these symptoms support these clinical reports of reduced severity of deficits. Videofluoroscopic results indicate patients with SMA Types II–IV typically have much lower rates of bolus airway entry (Banno et al., 2017; Cha et al., 2010; Hashizume et al., 2017; van den Engel-Hoek et al., 2009). Though a case report of a 25-year-old patient with SMA Type II revealed impaired laryngeal closure resulting in aspiration (Cha et al., 2010), other larger investigations have indicated none of their participants exhibited aspiration and 0%–26% of patients exhibited penetration (Banno et al., 2017; van den Engel-Hoek et al., 2009). Interpretation of these findings requires careful consideration to natural variants in deficits by SMA type, as well as findings from healthy, nondysphagic adults, which indicate 2% demonstrate aspiration and 9% demonstrate penetration on a videofluoroscopic swallow study (Garand et al., 2019). Discrepancies between these investigations may, in part, stem from differences in age of populations, procedural protocols, and analysis methods. More prevalent and different from healthy normal controls were observations of impairment in mastication and bolus clearance. Banno et al. (2017) found individuals with SMA Types III–IV exhibited significantly worse oral and pharyngeal bolus clearance resulting in pyriform and vallecular residue, as well as worse scores in mastication, epiglottic inversion, nasal regurgitation, and laryngeal closure than healthy controls (Atsuta et al., 2006). Similar findings were supported by Hashizume et al. (2017). Other reports indicate the source for deficits in pharyngeal clearance is related to deficits in upper esophageal sphincter opening (Suzukia et al., 2007; van den Engel-Hoek et al., 2008). Figure 4 provides a fluoroscopic image of these deficits in a patient with SMA Type III compared to a healthy normal counterpart.

Isolated case reports have indicated successful improvement in symptoms associated with impaired airway protection and pharyngeal clearance through rehabilitation and surgical techniques. Van den Engel-Hoek et al. (2008) reported improvement in coughing and choking episodes in a 5-year-old with SMA Type II through the modification of his intake regimen, including the consumption of puree solids, the use of a liquid wash, and the provision of a head band attached to his wheel chair to neutralize head posture. Cha et al. (2010) found beneficial effects in airway protection by training the supraglottic swallow maneuver in a 25-year-old with SMA Type II. Likewise, Suzukia et al. (2007) reported a 21-year-old patient with SMA Type II with this deficit resulting in clinically significant impairments. Successful treatment was achieved through the injection of five units of botulinum toxin to the cricopharyngeus muscle. Future longitudinal investigations using standardized metrics that allow quantification of these deficits are warranted.

*Mandibular range of motion and strength:* Many of the aforementioned deficits in chewing and swallowing solids have been linked to impairments in mandibular range of motion and strength (Austin et al., 2010; Granger et al., 1999; Kooi-van Es et al., 2020; van Bruggen et al., 2011, 2016; Wadman et al., 2014; Willig et al., 1994). Muscle contracture of the temporomandibular joint characterized by restriction in jaw range of motion is a common finding among all types of SMA, though the severity is correlated with SMA type. In an investigation measuring maximum mouth opening, Wadman et al. (2014) found 100% of patients with SMA Type I had reductions in the extent of active opening in comparison to 79% of patients with SMA Type II, 50% with SMA Type III, and 7% of patients with SMA Type IV. Similar trends were found in tracking the severity of this restriction, with some patients with SMA Type I exhibiting < 10 mm of opening between upper and lower incisors in contrast to > 50–60 mm among healthy individuals (Wadman et al., 2014). van Bruggen et al. (2011, 2016) demonstrated these restrictions also occurred in mandibular lateralization and protrusion.

Investigations examining the source of these impairments suggest atrophy of the lateral pterygoids, geniohyoid, and anterior belly of the digastric muscles may initially inhibit an individual's active mandibular range of motion (Wadman et al., 2014). However, this muscular mediated reduction in mobility transforms to a structurally induced restriction to even passive mouth opening attempts as a result of contracture of the temporomandibular joint (van Bruggen et al., 2011; Wadman et al., 2014). Adding to difficulties in chewing is atrophy of the masseter musculature (Wadman et al., 2014). The composite effect of the restrictions in range of motion and strength of closing contraction is a significant reduction in an individual's bite force (Granger et al., 1999). An investigation of these forces among individuals with SMA Types II and III indicates maximum bite force reductions as high as 56% of healthy peers with even greater reductions in their ability to sustain their maximum forces, which were 62% shorter than healthy peers (Granger et al., 1999). Morris et al. (2020) reported the ability to improve mandibular range of motion in a 32-Year-old patient with SMA Type II through the completion of hourly passive mandibular range of motion stretching using the TheraBite device over the course of 10 weeks. Likewise, Cha et al. (2010) reported similar improvements using “gentle joint mobilization” techniques targeted at the temporomandibular joint. Significance of these mechanical and muscular deficits has clear implications for mastication. Of equal importance to systemic health is the barrier they pose to an individual's ability to receive adequate oral care to prevent bacterial growth that fuels the pneumonia sequala.

*Craniofacial morphology:* Weakness of the head and neck musculature also has implications for morphological changes in craniofacial development. Generally speaking, SMA is associated with unique craniofacial features that set patients with SMA apart from healthy counterparts, a “myopathic facies.” This is characterized by excessive vertical development of the lower face and reduced vertical development of the upper face, leading to the appearance of long narrow jaws (Houston et al., 1994). Other unique attributes are specific to palatal and dental structures, where there is significantly greater maxillary than mandibular growth and proclamation of the upper incisors (Houston et al., 1994; van Bruggen et al., 2011). This results in the maxilla over jetting nearly 2 times the amount it should beyond the mandible (van Bruggen et al., 2011). The source of these inappropriate growth trajectories and structural proportions has been attributed primarily to weakness of facial, lingual, and labial musculature that, in typically developing children, apply tension to appropriately shape these developing structures (Houston et al., 1994; van Bruggen et al., 2011). Our work and that of others have also appreciated these morphological changes to include the presence of a high, narrow palatal groove, likely stemming from the lack of resting palatal pressure by the tongue (Enomoto et al., 2017; Grotto et al., 2016; Yu & Gao, 2019). These structural differences have been associated with reports of choking on solids and impaired mastication, likely indicating they too play a role in reducing an individual's ability to safely and efficiently eat solids. Along similar lines are the implications of skeletal malformations of the hip/femoral head and spine resulting from muscle weakness that are pervasive among individuals with SMA and require adaptive positioning for all aspects of daily living. While these adaptive positions are necessary, they often leave patients in suboptimal positions for oral intake that exacerbate oropharyngeal swallowing weakness.

### Theme 4: Dysphagia Outcomes With Disease-Modifying Therapies

Seven articles pertaining to dysphagia outcomes with disease-modifying therapies were identified and summarized in Table 3. Three of these articles reported dysphagia outcomes as part of a pharmaceutical trial, while the other four reported dysphagia outcomes of participants who had disease-modifying therapy outside a trial context.

**Table 3.** Details of investigations reporting dysphagia outcomes in patients with spinal muscular atrophy (SMA) receiving disease-modifying therapies.

Author	Year	SMA type	*N* ^a^	Disease-modifying therapy	Age at treatment^b^	Only baseline/adverse events^c^	Full pre- and postassessment^d^	Instrumental assessment	Method of assessment
Disease-modifying therapy trials									
de Vivo et al.	2019	I, II	25	Nusinersen	Asymptomatic 22 (3–42)	N	N	N	HINE-1: 3 = *good suck/swallow,* 1 = *poor suck/swallow,* 0 = *no suck/swallow*
Finkel et al.	2017	I	81	Nusinersen	Symptomatic 163 (52–242)	Y	N	N	Descriptive statistics of patients with (a) “swallowing or feeding difficulties” and (b) use of a gastrointestinal tube
Mendell et al.	2017	I	12	AVX-101	Symptomatic 102 (3–237)	N	N	N	1. Alternative nutrition 2. Descriptive: reported (a) “ability to swallow independently,” (b) “able to feed orally,” (c) “dysphagia,” and (d) pneumonia aspiration
Nontrial reports									
Kraszewski et al.	2018	I	1	Nusinersen	Asymptomatic 15 Days	N	Alternative nutrition Respiratory health	Full oral intake at 12 months with no nutritional support or respiratory compromise	
Kruse et al.	2020	II	2	Nusinersen	Not reported	Y	Bite force	Improvement in bite force posttreatment	
Matesanz et al.	2020	0	1	Nusinersen AVX-101	Symptomatic 14 days	N	Alternative nutrition	No reports of baseline performance beyond “weak suck” but indicate in posttreatment section that she “remains dependent on feeding tube” following receipt of both treatments. State best intake of up to 10 ml fluid and some puree at 7–8 months of age, however regression since	
van der Heul et al.	2020	I	5	Nusinersen	Asymptomatic Symptomatic 63 (3–218)	N	Clinical assessment VFSS Alternative nutrition	Initial improvement in sucking and swallowing posttreatment followed by decline at 8–12 months with functional with coughing, respiratory infections, silent aspiration, and need for alternative nutrition	

*Note.* N = no; Y = yes; HINE-1 = Hammersmith Infant Neurological Examination; VFSS = videofluoroscopic swallow study.

a Represents number of participants with SMA within the disease-modifying treatment arm where dysphagia outcomes were reported.

b Mean/median (range) days, unless otherwise reported.

c Indicates swallowing outcomes were not systematically collected but instead only reported in baseline or adverse events section.

d Full (pre and post) assessments require report of at least descriptive statistics at both time points and do not include clinical description of select patients.

*Spinraza (nusinersen):* The bleak outlook for patients with SMA underwent a complete transformation in 2016 with the FDA approval of the first disease-modifying therapy capable of stopping disease progression among all patients with SMA: Spinraza. Nusinersen is an antisense oligonucleotide that allows for *SMN2* to produce more of the functional SMN protein than it typically would and thereby facilitate motor neuron survival (Chiriboga et al., 2016; Passini et al., 2011). Due to its mechanism of action, administration requires injection directly into the cerebrospinal fluid via lumbar puncture. These injections occur every 2–4 weeks during the initial four loading doses, after which they occur every 4 months for the life span of the patient (de Vivo et al., 2019). Infant injections are typically provided without sedation to minimize repeated sedation risk; however, sedation frequently becomes necessary with increasing patient age. Research indicates beneficial treatment effects for nusinersen among all SMA Subtypes I–III, including survival; achievement of motor milestones such as head control, sitting, rolling over, and standing where previously trajectories only showed regression; and less adverse events such as acute respiratory failure and respiratory infections (de Vivo et al., 2019; Finkel et al., 2016, 2017; Mercuri et al., 2018). As nusinersen prevents the degeneration of neurons through SMN upregulation, it cannot facilitate the regeneration of those motor neurons already lost; hence, the magnitude of treatment effect is associated with the timing of treatment provision. For example, 61% of infants with SMA Type I who received nusinersen survived when they received their first dose at an average age of 5 months old (Finkel et al., 2017), whereas 100% of infants survived when they received their first dose at an average age of 3 weeks old (de Vivo et al., 2019). Remarkably, not only did the majority of infants who received their first dose before 6 weeks of age achieve motor milestones of sitting without support (100%), walking with assistance (92%), and walking alone (88%), most of the infants achieved these milestones within age-appropriate windows of development (de Vivo et al., 2019). The maintenance of these results following the completion of published reports is encouraging; however, much remains unknown regarding the long-term overall health of these patients, as the oldest to receive this treatment at the time of publication is 6 years of age (Yeo & Darras, 2020).

Although there is a growing body of literature regarding the effect of nusinersen on SMA motor function and survival, similar to natural history data, limited data are available regarding its effect on deglutition. This is in large part due to imprecise dysphagia definitions, the lack of sensitivity and validation of utilized measures, and the presence of baseline deficits. For example, in the initial sham-controlled nusinersen trial where treatment was given at an average age of 5 months, 51% of participants receiving nusinersen were reported to have “swallowing or feeding difficulties” prior to receiving treatment, with 9% requiring alternative nutrition. Following treatment, 11% who received nusinersen were reported to have “dysphagia” compared to 22% of participants in the control group. Specifications of the aforementioned definitions were not indicated, nor were posttreatment alternative nutrition rates, making interpretation of these results difficult (Finkel et al., 2017). Recent work by van der Heul et al. (2020) examining the effect of nusinersen on swallowing among five symptomatic infants who received treatment indicates an initial improvement in clinical feeding performance among some patients, followed by complete degradation and gastrostomy reliance by all patients. Videofluoroscopic swallow studies that were completed on four of these patients revealed silent aspiration among all participants (van der Heul et al., 2020).

More insight into potential swallowing effects can be gained by examining results from the investigation that examined treatment effect on presymptomatic infants (without gastrostomy tubes) who received nusinersen at an average age of 3 weeks old. In addition to the tracking need for gastrostomy tube, investigators measured swallowing using the 3-point sucking and swallowing scale that is part of the Hammersmith Infant Neurological Assessment (3 = *good sucking and swallowing,* 1 = *poor suck and/or swallow,* 0 = *no sucking reflex, no swallowing*). At an average of 34 months of age, 88% of participants were reported to have good sucking and swallowing on clinical evaluation, with the remaining 12% of patients scoring as having poor sucking and/or swallow, with gastrostomy tube dependence (de Vivo et al., 2019). Similar beneficial swallowing outcomes in presymptomatic patients with SMA Type I have been reported in case reports with the ability to maintain full oral intake without functional impairments (Kraszewski et al., 2018). While the ability to draw large conclusions on the integrity of sucking and swallowing abilities from this data set is greatly limited by the subjective clinical nature of this outcome, the continuation of full oral feeds for the majority of infants indicates promise compared to untreated natural history reports that indicate 100% of patients with SMA Type I required gastrostomy for feedings by 1 year of age (Birnkrant et al., 1998; Davis et al., 2014; Finkel, McDermott, et al., 2014). Trials investigating the effects of disease-modifying therapies, such as nusinersen on patients with SMA Type 0, have not yet been completed as a result of the severity of neuromuscular degeneration at birth. Isolated case reports do suggest nusinersen may have beneficial effects at sustaining life, as well as some gains in motor milestones in these patients; however, promise for improvement in already lost swallowing physiology and function is guarded, as evident by reports of continued deficits in oropharyngeal swallowing requiring complete dependence on alternative nutrition (Matesanz et al., 2020).

The only investigation examining the effect of nusinersen on oropharyngeal swallowing among patients with the less severe forms of SMA Type II was a pilot study completed by Kruse et al. (2020), which examined the effect of nusinersen on bite force. Investigators measured maximum bite force of two adults with SMA Type II using a piezoelectric force sensor before, during, and after nusinersen doses over the course of 1 year (Kruse et al., 2020). Results indicate a systematic significant increase in maximum bite force from baseline throughout the provision of the initial loading doses (Kruse et al., 2020). Future studies with larger sample sizes are necessary to further elucidate these physiological and corresponding functional chewing and swallowing effects.

*Zolgensma* (*AVXS-101; onasemnogene abeparvovec-xioi)*: Three years following approval of the first disease-modifying therapy for SMA came the approval of the second: Zolgensma. In contrast to nusinersen, which was approved for patients of all ages and SMA types, onasemnogene abeparvovec-xioi (hereafter referred to as *onasemnogene*) is strictly approved for those who are less than 2 years of age. Onasemnogene is a gene therapy that is provided through a one-time intravenous injection. The pharmaceutical works by using a virus to deliver a functional version of *SMN 1* gene to produce SMN protein, which is expressed throughout the body including motor neurons. The premise being that increased SMN levels improve cellular function and survival, and in the case of improved motor neuron health, this impacts muscle innervation and motor function in a positive manner (Al-Zaidy et al., 2019; Mendell et al., 2017). Results of the early efficacy study completed on symptomatic infants receiving treatment at 3.4 months of age indicate tremendously beneficial treatment effects, including survival at 20 months of age (100%), and improvement in motor function (Mendell et al., 2017). These included head control and unassisted sitting for 5 s (92% of participants), rolling over (75%), crawling, standing, and walking (17%; Mendell et al., 2017). Reports of swallowing outcomes were limited to clinical observation of swallowing to determine if a swallow was present or absent and assessment of a child's reliance on alternative nutritional support. These assessments indicate 92% of participants had a swallow present on clinical assessment, though 50% required alternative nutritional support (Mendell et al., 2017). While these results certainly hold promise, interpretation of outcomes between this investigation and those testing the effect of nusinersen requires consideration of the different study designs. Patients enrolled in the onasemnogene trial received treatment 1.5 months earlier than those in the initial nusinersen trials. Furthermore, comparison of baseline characteristics suggests those who received nusinersen may have had more severe swallowing deficits, with 51% who received nusinersen reporting baseline swallowing or feeding difficulties compared to 41.7% in the onasemnogene trial. Future investigations examining the difference in swallowing effects of these pharmaceutical options on comparable samples of pre- and postsymptomatic patients are warranted to further clarify differences in anticipated deglutition outcomes.

## Discussion

As the SMA community moves forward in this new era with effective SMA treatments now available and more on the horizon, rapid data dissemination and clinical management adaptations are critical to optimizing patient outcomes. Such practice is evident in the addition of routine SMA screening to the newborn screening panel in many states throughout the United States and internationally. This has allowed earlier diagnosis in many instances, with the goal of initiating treatment before irreversible motor neuron degeneration has occurred to maximize patient outcomes. It is important to note, however, that, while this new frontier of disease-modifying therapies holds tremendous promise for improving swallowing outcomes, gaps in the summarized articles indicate a paucity of investigations that systematically evaluate the effect of these therapies on both physiological and functional swallowing metrics, which leaves the clinical community without the necessary evidence to guide their dysphagia care. Those investigations reporting on oropharyngeal swallowing outcomes rarely did so using a systematic approach. Pre- and postswallowing outcomes were seldom reported in a uniform manner, without any of the investigations reporting validated assessment metrics. It is notable that this is not the case for measures of gross systemic function, such as the Hammersmith Infant Neurological Examination, the Bayley Scales of Infant and Toddler Development Gross Motor subtest, and the Children's Hospital of Philadelphia Infant Test of Neuromuscular Disorders, which have been regularly implemented across SMA trials and other investigations (de Vivo et al., 2019; Finkel et al., 2016, 2017; Mendell et al., 2017). With the recent establishment of valid and reliable dysphagia metrics (Martin-Harris et al., 2018; K. E. McGrattan et al., 2020; Serel Arslan et al., 2018), the key step in integrating these metrics into the clinical and scientific arena is the involvement of the speech-language pathologist on the interdisciplinary neuromuscular clinical care and research teams.

What our clinical focus article clarified, however, is that patients who do not receive disease-modifying therapy consistently exhibit clinically significant deficits in oropharyngeal swallow function. In patients with SMA Type I, this is characterized by a rapid degeneration of oropharyngeal swallowing that leave children dependent on alternative nutrition and secretion management techniques for survival (Birnkrant et al., 1998; Choi et al., 2020; Davis et al., 2014; Finkel, McDermott, et al., 2014). While individuals with the less severe forms of SMA Types II–IV exhibit less severe deficits, they remain clinically significant as characterized by prolonged mealtimes, trismus, deficits in mastication, and choking in patient-reported outcomes (Chen et al., 2012; Granger et al., 1999; Messina et al., 2008). Literature is emerging that outline promising methods that may alleviate these symptoms such as the use of compensatory techniques, such as modification of diets and eating postures (van den Engel-Hoek et al., 2008), or direct approaches, such as surgery or passive stretching of the temporomandibular joint (Morris et al., 2020; Suzukia et al., 2007). While promising, it is critical to keep in mind that these investigations are limited to case reports, with large controlled investigations lacking. A key step in filling this gap and establishing effective dysphagia interventions, especially among untreated SMA Types I–IV who do not always receive disease-modifying treatment, is understanding the physiological target. Continuing work that outlines the physiological sources for these functional oropharyngeal swallowing impairments is a key step in moving forward to help alleviate patients of these deficits.

While the articles summarized in this clinical focus article provide clinicians with the foundational knowledge pertinent to SMA dysphagia care, it is important to keep in mind that the breadth and the rigor of the identified articles were not evaluated using a standardized or systematic review method. As such, this article does not provide a comprehensive list of all articles relating to the aforementioned domains. Likewise, due to the paucity of literature within the deglutition domain, articles reporting single-subject study results were included in this article. Results of these investigations should be interpreted cautiously, as such a design does not allow for generalizable results.

As the field moves forward, further areas of investigation and clinical adaptation surround the utilization of combination disease-modifying therapies. At the time of this article's publication, there are currently three FDA-approved disease-modifying therapies for the treatment of SMA. While no controlled trials testing the effect of combination therapy have been conducted to date, this practice is occurring across many centers and warrants investigation. Likewise, with the diversion from the previously utilized palliative aerodigestive management approaches, where evaluation and decision making around placement of gastrostomy tubes was at the core of SMA clinical care, the role of the dysphagia expert on the neuromuscular team has become even more critical. Modifications to SMA care guidelines have called for regular instrumental assessment for newly diagnosed patients and for older individuals experiencing complications or simply gradual disease progression. In the era of disease-modifying therapies, historic clinical classifications, as those described in Table 1, are changing, and disease trajectories are less clear. That said, aerodigestive risks cannot be dismissed, and the need for vigilance is, perhaps, more important. We must appreciate that individuals with SMA may have different responses to different treatments. Furthermore, we must acknowledge that motor neurons and corresponding functionality lost before treatment will not be resurrected. Thus, setting expectations and mitigating risks through regular assessment of dysphagia, provision of dysphagia therapies, and parallel discussions about nutrition and hydration options remain essential. Optimizing health and pursuit of SMA treatments are not mutually exclusive, rather synergistic.
